# Critical Thinking and Clinical Reasoning in Undergraduate Medical Course: A Mixed-Methods Study in a Medical College in Kolkata, West Bengal, India

**DOI:** 10.12688/f1000research.146009.1

**Published:** 2024-04-11

**Authors:** Dipta Kanti Mukhopadhyay, Sonali G. Choudhari

**Affiliations:** 1 School of Higher Education & Research, Datta Meghe Institute of Higher Education & Research, Sawangi (Meghe), Maharashtra, 442107, India; 2 Community Medicine, College of Medicine and Sagore Dutta Hospital, Kolkata, West Bengal, 700058, India; 3 Community Medicine, Jawaharlal Nehru Medical College, Sawangi (Meghe), Maharashtra, 442107, India

**Keywords:** Critical thinking, Clinical reasoning, Medical Students, Education, Medical, Undergraduate

## Abstract

Critical thinking is considered as the essential set of skills for medical practice, particularly during emergencies. However, there is lack of conceptual clarity around it and it was not explicitly included in the undergraduate medical curriculum in India.

The present study has been planned to assess the critical thinking disposition and clinical reasoning skills among medical undergraduate students in a medical college in West Bengal, India. The perceived definition and attributes of critical thinking in medical education will be explored and the contexts where application of critical thinking skills may be crucial for medical practice will be identified.

The content validity index, test-retest agreement; internal consistency and construct validity of the Critical Thinking Disposition Assessment Questionnaire (CTDAQ) will be assessed through step-by-step procedure. CTDAQ and the case-based objective-type questions for the clinical reasoning skills will be applied to around 200 medical undergraduate students. Their perception and experience on critical thinking in medical education will be assessed with structured open-ended questions. In-depth interviews with medical teachers of the second and third phases of undergraduate medical curriculum will be conducted to assess their perception and experiences on critical thinking.

The quantitative analysis will be conducted with MS excel and R software using the relevant packages. The qualitative data will be transcribed and translated in English, close to the participants’ verbatim. The thematic analysis will be conducted with inductive coding and memoing.

The study will be conducted maintaining ethical standards for epidemiological studies.

The present study, one of the first a few studies in India, will help to meet the conceptual gap in understanding the attributes of critical thinking, its association with clinical reasoning and the contexts of preferred application in medical practice.

## Introduction

Critical thinking, the ability to think clearly and rationally about what to do or what to believe, is essential for the practice of medicine. ^1^ Although it is not a new concept in medical education, most of the published literature on critical thinking in the healthcare setting concerns nursing personnel. ^1^

**Figure 1.**
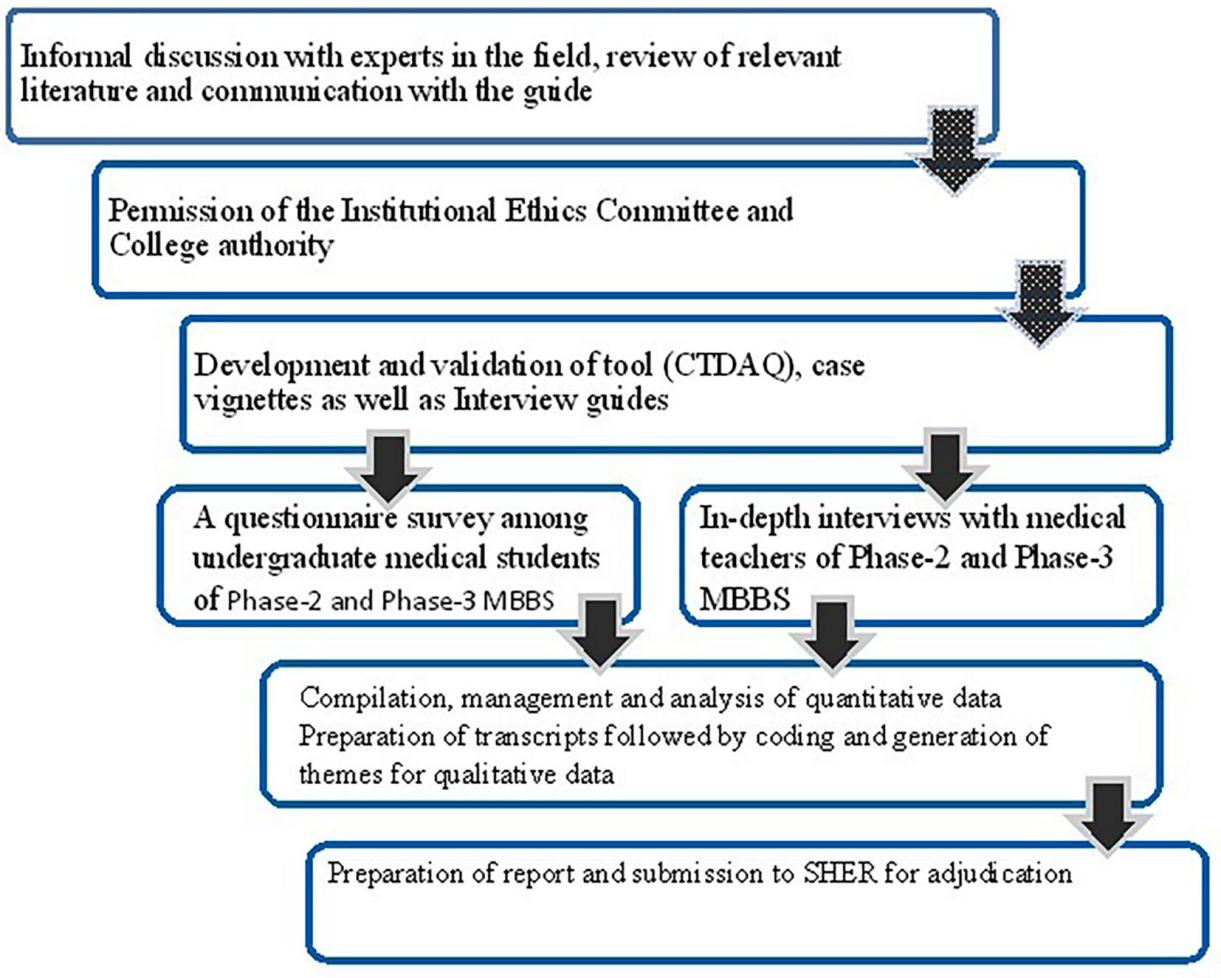
Flowchart showing the activity planning for the study.

Acquiring critical thinking skills is crucial for the practice of medicine. Doctors are supposed to make effective decisions in both well-defined and ill-defined emergencies. The inability to make appropriate decisions in ill-defined emergencies may lead to untoward incidents and affect the reputation of the healthcare facility and career of the individual doctor. ^2^ When encountering an ill-defined emergency, one of the primary reasons for difficulties is the inability to think critically. ^3^ Healthcare is prone to diagnostic and management errors. ^3^ Knowledge and cognitive processing skills are interrelated and interdependent, which in turn are associated with conceptual understanding and metacognition. ^3^ These skills, commonly referred as clinical reasoning skills, help in problem-solving based on the principles during unfamiliar and novel scenarios. ^3^ Strict adherence to standard operating procedures will help minimize errors in data collection and decision making in well-defined medical problems, but is not an effective strategy to stimulate critical thinking and creativity in undefined, resource-constrained settings. ^3^^,^^4^ Around one-third of problems in healthcare settings worldwide result from diagnostic errors. ^2^

Training in clinical reasoning and critical thinking may provide a solution to this problem, at least in part. Critical thinking is not explicitly included within the ambits of core competency in medical curricula in most cases. ^1^^,^^2^^,^^5^ Now, the time requires that teaching and learning critical thinking skills should be explicitly considered in the curriculum for medical undergraduates. However, critical thinking and its implications in undergraduate medical education suffer from a lack of conceptual clarity. ^6^^,^^7^ There are certain questions that remain unresolved: what is meant by critical thinking to medical educators and students, where in the medical curriculum does it appear, when, and how can it be inculcated, particularly in resource-constrained setting? ^6^^,^^7^

The Watson and Glaser Critical Thinking Appraisal and the California Critical Thinking Disposition Instrument are commonly used to assess critical thinking in different settings, primarily in Western culture. ^8^ Several questionnaires were developed to assess critical thinking disposition among medical professionals in Asian cultures. ^8^^,^^9^ Two of these are the 19-item Critical Thinking Disposition Assessment (CTDA) questionnaire in English and 27-item Yoon’s Critical Thinking Disposition (YCTD) Instrument. As there is transcultural variation in the meanings of different constructs, the questionnaire to be used needs to be validated in the Indian setting. ^8^^,^^9^

In this background, the present study is planned in a Medical College in Kolkata, West Bengal, India, with the following objectives:

1. To develop and validate a questionnaire to assess critical thinking disposition among undergraduate medical students
2. To assess the critical thinking disposition among medical undergraduates at different levels of the course
3. To assess the clinical reasoning skills among medical undergraduates at different levels of the course
4. To find out the correlation between critical thinking and clinical reasoning among medical undergraduate students
5. To explore the perceptions and experiences of undergraduate medical students regarding critical thinking in medical education
6. To explore the perceptions and experiences of medical teachers regarding critical thinking in medical practice and education

## Methods

### Study type and design

This will be a descriptive, cross-sectional study using mixed-methods approach. Mixed-methods research allows researchers the opportunity to gain a more meaningful understanding of the problems and answer questions that may have been partially answered had quantitative or qualitative data alone. ^10^^,^^11^

The mixed-methods approach will help to triangulate different systematic measures through quantitative and qualitative approaches to provide a comprehensive scenario on critical thinking in medical education. ^12^ Critical thinking disposition and clinical reasoning skills will be quantitatively measured. However, to enhance the critical thinking and clinical reasoning skills among undergraduate medical students, it is imperative to understand the perceptions and experiences about what is meant by critical thinking and what are its essential attributes, as well as where and how training on those skills be placed in the undergraduate medical curriculum. This will be explored using qualitative methods.

The collection of both quantitative and qualitative data will be conducted simultaneously.

### Study setting

The study will be conducted at a Medical College at the outskirts of the Kolkata Metropolitan area in the district of North 24 Parganas of West Bengal, India.

### Study duration

The total duration of the study will be ten months. First two months will be used for preparatory work including development and validation of tool. Next four months will be used for collection of data from study participants. The last four months will be utilized for data entry, analysis and report writing.

### Study population

1. The tool will be validated among undergraduate medical students *(for objective-1)*
2. The students enrolled in the second and third phases of undergraduate medical course during the data collection period in the referred college ( *for objectives2-5*)
3. The Medical Teachers of the referred college who are imparting teaching during the second and third phases of undergraduate medical course ( *for objective 6*)

### Sample size and sampling method

For step-by-step validation of the questionnaire, the required sample size was as per the guidelines on educational research. ^13^^,^^14^

The convenience sampling method will be used to approach all students (~ 125 per year-batch) enrolled in the second and third phases of MBBS at the College of Medicine and Sagore Dutta Hospital, Kolkata. Assuming a 20% non-response rate, the approximate number of respondents will be 200.

Around 20-25 Medical Teachers, depending on data saturation, attached to various departments of the College of Medicine and Sagore Dutta Hospital, Kolkata, imparting teaching and training during the second and third phases of MBBS, will be included for in-depth interviews through purposive stratified sampling.

### Tools & techniques

1. A self-administered questionnaire with both closed- and open-ended questions will be administered to the students.
2. Case vignettes with follow-up questions will be applied to the students
3. Interview Guide for Medical Teachers of the second and third phases ^6^^,^^15^

### Method of data collection

The first section of the questionnaire will contain an informed consent form. The students will respond to the next section only after providing their consent. The second section will consist of anonymous structured questions on the socio-demographic and individual characteristics of the students.

The third section will consist of questions on different domains of Critical Thinking Disposition (CTD) and a few open-ended questions. ^8^^,^^9^^,^^16^ The open-ended questions will explore the perceptions and experiences of medical students regarding critical thinking skills in medical education. The validated questionnaire will be used to assess critical thinking among undergraduate medical students in different phases.

The fourth section will contain case vignettes with follow-up questions to assess clinical reasoning skills. With the involvement of subject material experts, a series of case vignettes will be prepared with a case scenario followed by some questions to assess the clinical reasoning skills from five broad clinical specialties (Internal Medicine, Community Medicine, General Surgery, Pediatrics and Gynecology & Obstetrics) of the undergraduate medical curriculum. These case vignettes with follow-up supply type objective questions will be applied to assess the clinical reasoning skills of the students in each selected subject.

Both the authors were trained in qualitative research techniques. After obtaining written informed consent, the first author will conduct in-depth interview with audio recording with medical teachers from the selected medical college in a place and time of mutual convenience, with the help of the interview guide. After collecting socio-demographic and individual information about the teacher, their perceptions and experiences of critical thinking skills in medical practice and education will be explored. They will be requested to identify the context in medical practice where critical thinking skills can play a crucial role. ^6^^,^^15^

### Outcome variables

1. Critical Thinking Disposition: It will be measured using the domain-wise score and the total score. A higher score reflects a higher critical thinking disposition of the students. Students will be categorized based on quartile values: those with scores greater than the third quartile value will be considered to have a high critical thinking disposition, those within the third and first quartiles will be considered to have an average, and those below the first quartile will be considered to have low critical thinking disposition.
2. Clinical Reasoning Skills: It will be assessed using the total score obtained by the individual students from the case-based questions. A higher score reflects higher clinical reasoning skills. Students will be categorized based on quartile values: those with scores more than the third quartile value will be considered to have high clinical reasoning skills, those within the third and first quartiles will be considered to have average, and those below the first quartile will be considered to have low clinical reasoning skills.
3. The perceptions and experiences of students and teachers on critical thinking skills in medical education and practice under
  a) Conceptual definition of critical thinking
  b) The essential attributes of critical thinking
  c) The importance of critical thinking skills in medical practice
  d) Contexts in medical practice, where the application of critical thinking skills may be crucial in terms of outcomes

### Plan for data analysis

The Critical Thinking Disposition Assessment Questionnaire (CTDAQ) will be developed and tested for content validity with the help of experts from the field of Psychiatry, Psychology and Medical Education. Item-wise and total content validity indices will be calculated in MS Excel. It will first be applied to 25 first-year students twice, with a gap of three weeks to check test-retest agreement with weighted kappa statistics. It will then be applied to 150 MBBS students, other than the study population, to check internal consistency with Cronbach’s alpha and construct validity through exploratory principal component analysis with varimax rotation. ^13^^,^^14^ All analysis will be conducted using different packages ( *kappaGUI, cronbach* and *prcomp*) of open-source ‘R’ 4.3.1 software. ^17^

The data gathered through the questionnaire survey will be entered into an MS Excel spreadsheet and tested for consistency. The individual items of the questionnaire will be rated on a five-point Likert scale, ranging from 1 ( *strong disagreement*) to 5 ( *strong agreement*). The scores of all items under a specific domain will be added to obtain the domain-wise score. Domain-wise median (IQR) scores will be calculated for each phase and compared. Case-based questions will be marked according to the protocol designed by subject material experts. All the marks attained collectively in all case vignettes will be added to obtain total marks. The central tendency and dispersion will be expressed as the median and IQR of the marks.

Spearman correlation will be used to examine the relationship between the total scores of the critical thinking disposition assessment questionnaire and the total score from the test of clinical reasoning skills.

In-depth interviews will be transcribed from audio tape and field notes and then translated into English by the first author, close to verbatim within 48 hours of the interview. Participant confirmation will be ensured by Medical Teachers individually. The codes will be generated by two authors separately through inductive reasoning. They will be compared and contrasted, and discrepancies will be sorted out through discussion. Then, the codes will be collated with the help of memoing to generate categories and themes. The audit-trail of the analytic pathways will be maintained.

### Ethics

The study will strictly adhere to the Declaration of Helsinki and follow the standards of observational study. The study obtained approval from the Institutional Ethics Committee, College of Medicine and Sagore Dutta Hospital, Kolkata-700058 (Registration. No. ECR/1210/Inst/WB/2019/RR-22) with Approval No. CMSDH/IEC/107/12-2023; date: 04.12.2023.

Written informed consent will be obtained from each participant after explaining the purpose, procedure, and probable outcome of the study, ensuring confidentiality of data, voluntary participation, and right to withdraw at any point in time. The format was approved by the concerned Institutional Ethics Committee.

The transcripts will be kept under custody of the authors to maintain anonymity and confidentiality. The same principle will be applied to the quantitative and qualitative databases.

### Dissemination

The study is planned as a dissertation for Masters in Health Professional Education (MHPE) and will be submitted to the university. The results of the study may be published in medical/health journals.

### Study status

The study is yet to start.

## Data Availability

No data are associated with this article.

## Software availability

The data will be analyzed using open-source R software, which is available from https://cran.r-project.org/bin/windows/base/. ^17^ The references were managed using Zotero software ( https://www.zotero.org/download/). ^18^
