# Supplementary material for: Critical Thinking and Clinical Reasoning in Undergraduate Medical Course: A Mixed-Methods Study in a Medical College in Kolkata, West Bengal, India
Source: F1000Res. 2024 Apr 11;13:259. [Version 1] doi: 10.12688/f1000research.146009.1 (PMC11109580; doi:10.12688/f1000research.146009.1)
Supplement: Peer review history

# Reviewer report: Reviewer response for version 1

**DOI:** 10.5256/f1000research.160038.r271974

**Published:** 2024-05-21

**Authors:** Sanhita Mukherjee

**Affiliations:** 1 Rampurhat Government Medical College, Rampurhat, West Bengal, India

APPROVED.

 Critical thinking is an essential cognitive skill for every individual but is a crucial component for healthcare professionals such as doctors. This helps the medical students to analyze the information critically and then apply that to the existing information. Critical thinking skill is considered a cornerstone for teaching and training medical students so as to maintain clinical competence and medical professionalism.

 This study nicely tries to assess the the critical thinking disposition and clinical reasoning skills among medical undergraduate students. This is an excellent attempt to develop and validate a questionnaire to assess critical thinking skill.

 I found a flawless method by which they are trying to develop the Critical Thinking Disposition Assessment Questionnaire (CTDAQ).

 Traditionally, higher education is thought to produce career thinkers equipped with the knowledge and intellectual abilities. However, there is a growing awareness that many students do not have expected professional abilities. Therefore, the higher education system needs development of these thinking abilities in curriculum. Specially in medical field to reach correct diagnosis one should not only depend on knowledge but also on this CRT in order to reduce the pain and harassment of the patient. CRT should be taught and assessed in undergraduate medical students in order to make them competent before they start their clinical practice.

 This study is a visionary effort toward this direction which is very rare in Indian medical schools.

 Although CT is influenced by many educational and sociological parameters, curriculum is the most important parameter every student must deal with during academic studies. If a curriculum is based on CT skills, it directs learners toward disposition to CT. This study shows a crucial step towards  inclusion of CRT in undergraduate medical curriculum in India.

 Perception of students on CRT will also help to understand student's point of view on it. Students of this era of social media and AI are more practical and tech savvy. The patients and their relatives are also open to the information domain. Previously in India ignorance of patient and patient party about medical science made the job of the clinicians easier. Nowadays in order to face well informed patient and to communicate with them CRT is essential.

 Hence I approve this study.

Is the study design appropriate for the research question?

Yes

Is the rationale for, and objectives of, the study clearly described?

Yes

Are sufficient details of the methods provided to allow replication by others?

Yes

Are the datasets clearly presented in a useable and accessible format?

Yes

Reviewer Expertise:

Medical Education

I confirm that I have read this submission and believe that I have an appropriate level of expertise to confirm that it is of an acceptable scientific standard.

# Reviewer report: Reviewer response for version 1

**DOI:** 10.5256/f1000research.160038.r271971

**Published:** 2024-05-15

**Authors:** Ananya Mandal

**Affiliations:** 1 Nil Ratan Sircar Medical College and Hospital, Kolkata, India

I found this study proposal very intriguing. Moving head of rote learning that used to be the cornerstone of medical education critical thinking can allow students to be better prepared for their lives as doctors. This is however not constructively taught to the students. As the authors have mentioned an in depth look at the methodology of such training for medical undergraduates is essential.

 Their planning involves content validity index, test-retest agreement; internal consistency and construct validity of the Critical Thinking Disposition Assessment Questionnaire (CTDAQ). This will provide a scientifically backed method of development of the module that may help in future teaching. Also their suggestion of using case based scenarios for the student questionnaire seems appropriate for fulfilling their ultimate objective.

 Another good part of their methodology is the use of in depth interviews of the medical teachers to assess their perception and experiences on critical thinking. The primary stakeholders of the system being the students and teachers, this seems to be vital.

 As such the plan for analysis doesn’t warrant a comment as it is well thought out and detailed.

 Overall, the protocol seems to be well planned and thought out. The study has not been conducted yet so data is not available for review. As such study methodology appears to be complete and logical.

**Comment:** Approved

Is the study design appropriate for the research question?

Yes

Is the rationale for, and objectives of, the study clearly described?

Yes

Are sufficient details of the methods provided to allow replication by others?

Partly

Are the datasets clearly presented in a useable and accessible format?

Not applicable

Reviewer Expertise:

Pharmacology, Medical Education, Clinical trials, Drug utilization, Rational drug use, Antimicrobial sensitivity and resistance

I confirm that I have read this submission and believe that I have an appropriate level of expertise to confirm that it is of an acceptable scientific standard.

# Reviewer report: Reviewer response for version 1

**DOI:** 10.5256/f1000research.160038.r271966

**Published:** 2024-05-09

**Authors:** Samy A Azer

**Affiliations:** 1 King Saud University, Riyadh, Riyadh Province, Saudi Arabia

Critical Thinking and Clinical Reasoning in Undergraduate Medical Course: A Mixed-Methods Study in a Medical College in Kolkata, West Bengal, India

 STUDY PROTOCOL

 Thank you for inviting me to review the above-titled manuscript. While the topic is intriguing, the manuscript is marred by a lack of focus, unclear research questions, and insufficient details.

 ABSTRACT- 1) Too many objectives. You must have a focus. 2) State your research question. 3) What did you use as a research method? What tools did you use? 4) We do not start methods by mentioning validity and reliability issues- We need to know what type of method was used to answer the research question and what tools were used. 5) What is "test-retest agreement"? "construct validity"- mentioned twice but never explained how these will be considered. 6) Who are the participants - include inclusion and exclusion criteria. 7) Which questionnaire data? Is this a validated questionnaire from the literature, or will the authors create it? 8) Statistical tests to be used is missing. 

 English editing is required for the whole manuscript. 

 INTRODUCTION- 1) Definition given is incomplete and not well addressed; please read the literature (first three lines). 2) "well-defined and ill-defined emergencies" - Why? and what do you mean? 3) Is this only about an emergency? Emergency has been repeated but not in the title or the abstract. Is critical thinking in medicine limited to Emergencies? 4) Define critical thinking, state its scope, and give examples. 5) Figure - "In-depth interviews". Why? Why not focus groups? You must explain and justify the purpose. 

 Need to know where in the course and which subjects, what situations in small group learning students experience critical thinking. 

 METHODS- 1) What do you mean by mixed methods - explain what will be qualitative and what will be quantitative, regarding research design, methods used, data collected, data analysis, etc. 2) Add citations and references to methods. 3) Study Population - Which tools? 3) What is the sample size and sample power? Explain the basis for your calculations, the equation to be used and give the exact number of sample sizes and sample types. 4) Several parts were not clearly written

 Plamn for data analysis- Should be edited, organised and focused. Avoid contradictions- Earlier, the author mentioned applying for ethical approval, then here they say, "The study has obtained ethical approval." - which one should we believe? 

 References - the authors should read and cite proper literature in Academic Medicine, Medical Education, Medical Teacher, BMC Medical Education, Teaching and Learning in Medicine, Journal of Surgical Education. 

 Ref 16- too old

 Ref 17- Should be replaced

 Ref 18- Not a reference- Should be replaced

Is the study design appropriate for the research question?

Partly

Is the rationale for, and objectives of, the study clearly described?

Partly

Are sufficient details of the methods provided to allow replication by others?

Partly

Are the datasets clearly presented in a useable and accessible format?

Not applicable

Reviewer Expertise:

I am professor of medical education for over 15 years. I have expertise in medical education, research methods, public health, and gastroenterology. I have over 120 published research in top journals and 5 textbooks.

I confirm that I have read this submission and believe that I have an appropriate level of expertise to state that I do not consider it to be of an acceptable scientific standard, for reasons outlined above.
